# Research Models and Gene Augmentation Therapy for *CRB1* Retinal Dystrophies

**DOI:** 10.3389/fnins.2020.00860

**Published:** 2020-08-14

**Authors:** Nanda Boon, Jan Wijnholds, Lucie P. Pellissier

**Affiliations:** ^1^Department of Ophthalmology, Leiden University Medical Center, Leiden, Netherlands; ^2^The Netherlands Institute for Neuroscience, Royal Netherlands Academy of Arts and Sciences (KNAW), Amsterdam, Netherlands; ^3^Biology and Bioinformatics of Signalling Systems, Physiologie de la Reproduction et des Comportements INRAE UMR 0085, CNRS UMR 7247, Université de Tours, IFCE, Nouzilly, France

**Keywords:** retinitis pigmentosa, leber congenital amaurosis, crumbs homolog 1, gene therapy, mouse model

## Abstract

Retinitis pigmentosa (RP) and Leber congenital amaurosis (LCA) are inherited degenerative retinal dystrophies with vision loss that ultimately lead to blindness. Several genes have been shown to be involved in early onset retinal dystrophies, including *CRB1* and *RPE65*. Gene therapy recently became available for young RP patients with variations in the *RPE65* gene. Current research programs test adeno-associated viral gene augmentation or editing therapy vectors on various disease models mimicking the disease in patients. These include several animal and emerging human-derived models, such as human-induced pluripotent stem cell (hiPSC)-derived retinal organoids or hiPSC-derived retinal pigment epithelium (RPE), and human donor retinal explants. Variations in the *CRB1* gene are a major cause for early onset autosomal recessive RP with patients suffering from visual impairment before their adolescence and for LCA with newborns experiencing severe visual impairment within the first months of life. These patients cannot benefit yet from an available gene therapy treatment. In this review, we will discuss the recent advances, advantages and disadvantages of different *CRB1* human and animal retinal degeneration models. In addition, we will describe novel therapeutic tools that have been developed, which could potentially be used for retinal gene augmentation therapy for RP patients with variations in the *CRB1* gene.

## CRB Family Members

**FIGURE 1:**
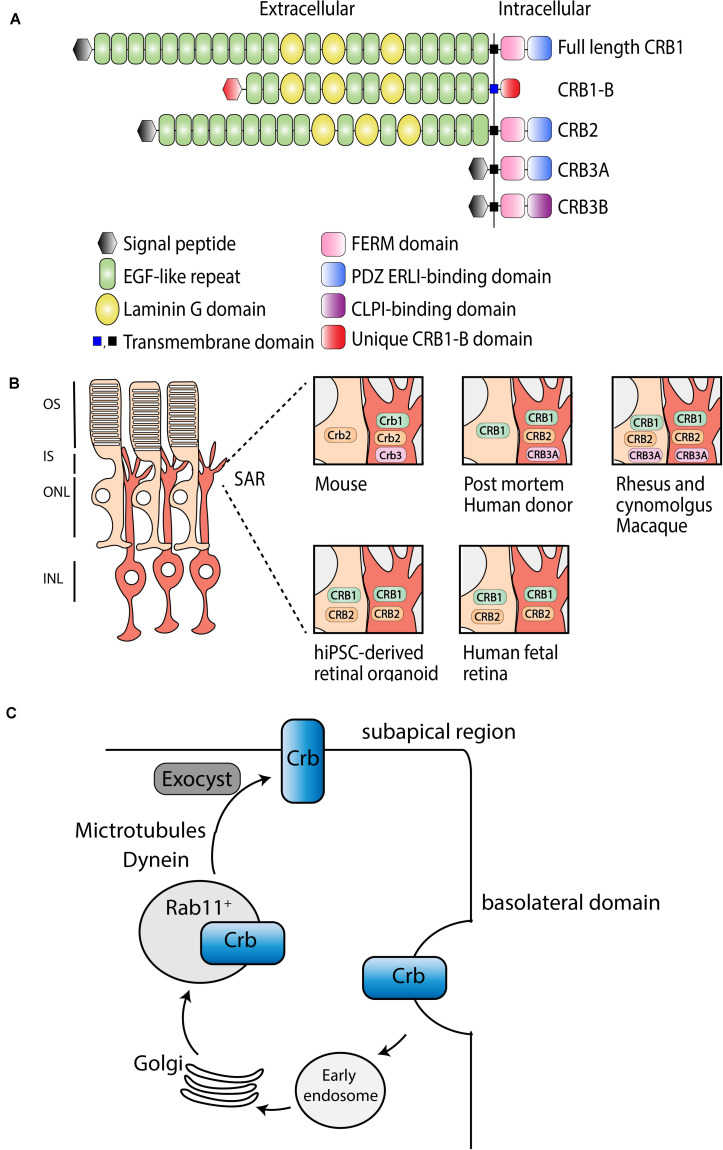
Schematic representation of CRB subcellular localization and proposed trafficking mechanism. **(A)** Schematic overview of full length CRB1 (CRB1-A), CRB1-B, CRB2, CRB3A, and CRB3B proteins. **(B)** Subcellular localization of CRB1, CRB2, and CRB3A in the retina of mouse, post mortem human donor, macaque, hiPSC, and human fetal retina. **(C)** Trafficking mechanism in *Drosophila* suggested to be conserved among species, adapted from Aguilar-Aragon et al. (2020).

Crumbs (Crb) is a large transmembrane protein initially discovered at the apical membrane of *Drosophila* epithelial cells (Tepass et al., 1990). Several years later, it was found that mutations in a human homolog of the *Drosophila melanogaster* protein crumbs, denoted as CRB1 (Crumbs homolog 1), was involved in retinal dystrophies in humans (Den Hollander et al., 1999). The human *CRB1* gene is mapped to chromosome 1q31.3, and contains 12 exons, has 12 identified transcript variants so far, three CRB family members, and over 210 kb genomic DNA (Den Hollander et al., 1999)^1^. Canonical CRB1 is, like its *Drosophila* homolog, a large transmembrane protein consisting of multiple epidermal growth factor (EGF) and laminin-globular like domains in its extracellular N-terminus (Figure 1A). The intracellular C-terminal domain contains a FERM and a conserved glutamic acid-arginine-leucine-isoleucine (ERLI) PDZ binding motives. An alternative transcript of CRB1, *CRB1-B*, was recently described and suggested to have significant extracellular domain overlap with canonical CRB1 while bearing unique 5′ and 3′ domains (Ray et al., 2020). In mammals, CRB1 is a member of the Crumbs family together with CRB2 and CRB3 (Figure 1A). CRB2 displays almost the same protein structure as CRB1, except a depletion of four EGF domains. CRB3A lacks the entire typical extracellular domain but contains the transmembrane domain juxtaposed to the intracellular part with the FERM-binding motif and a ERLI PDZ sequence. A second protein (isoform CRB3B) arises from the same *CRB3* gene due to alternate splicing of the last exon, resulting in a different C-terminus with a cysteine-leucine-proline-isoleucine (CLPI) amino acid sequence, and thus lacks the PDZ domain (Fan et al., 2007; Margolis, 2018). Interestingly, the CRB3B isoform is found in mammals, but not in zebrafish or *Drosophila* (Fan et al., 2007). Further details about CRB isoform details can be found in Quinn et al. (2017).

## CRB Localization in the Retina

In mammalian tissue CRB1 and CRB2 are predominantly expressed in the retina, however, CRB2 expression is also found in other tissues such as in kidney podocytes, in the subventricular zone of the brain, and in the spinal cord (Ebarasi et al., 2015; Slavotinek et al., 2015; Dudok et al., 2016; Tait et al., 2020). Within the retina, CRB proteins are localized at the subapical region (SAR) above the adherence junctions between photoreceptor and Müller glial cells (MGCs), multiple photoreceptor cells (PRCs), or between multiple MGCs (Pellikka et al., 2002; van de Pavert et al., 2004). In addition, unlike CRB1, CRB2 also localizes in the retinal pigment epithelium (RPE). Defining the subcellular localization is essential to understand the function of CRB proteins (Figure 1B). Mouse studies have shown that full length Crb1 protein is exclusively present in MGC at the SAR while Crb2 and Crb3 are present in both PRC and MGC (van Rossum et al., 2006). Serial tangential cryosectioning of the retina followed by western blotting displays *Crb1-B* transcript expression in outer segments of PRCs in mice (Ray et al., 2020). The first ultrastructural data in postmortem human retina revealed a different localization, where CRB2 is located in MGC at the SAR and at vesicles in photoreceptor inner segments, whereas CRB1 is located in both MGC and PRC at the SAR (Pellissier et al., 2014b, 2015). CRB3A was found in microvilli of MGC at the SAR and in inner segments of PRC (Pellissier et al., 2014b, 2015). However, recently, it was shown that CRB1 and CRB2 are located in MGC and PRC at the SAR in the second trimester of human fetal retina and in human iPSC-derived retinal organoids, whereas in the first trimester only CRB2 was detected (Quinn et al., 2019a). Single-cell RNA sequencing data of human fetal retina and RPE confirms that CRB1 is present in retinal progenitor cells and MGC but not in RPE, which is in accordance with mouse versus primate localization studies (Hu et al., 2019). In addition, similar localization for CRB1, CRB2, and CRB3A were detected in rhesus and cynomolgus macaques (Quinn et al., 2019b). CRB3A was also detected in the inner retina and RPE of both rhesus and cynomolgus macaques (Quinn et al., 2019b). These recent data suggest that, in humans, CRB2 might also be present at the SAR membranes in PRC rather than only in vesicles of photoreceptor inner segments. The discrepancies in CRB2 pattern at the SAR observed in postmortem human retinas versus monkeys, fetal and retinal organoids could be explained by the age of the donors studied, the quality of the samples (processed within 48 h after death), or by technical issues. We could speculate that CRB2 may have a different location in human aged retinas, CRB2 at the SAR might have not been detected, or CRB2 might have been endocytosed from the PRC plasma membrane following donor death. Additional experiments with fresh human retina defining the subcellular localization of CRB1 and CRB2 could potentially resolve these differences. In summary, according to all these evidences, we hypothesize that in primates, including humans, CRB1 and CRB2 are located in both cell types at the SAR. Therefore, both MGC and PRC should be targeted to prevent retinal degeneration in RP patients.

Recently, Crb trafficking to the correct apical location in *Drosophila* epithelium has been further investigated (Figure 1C; Li et al., 2007; Pocha et al., 2011; Kraut and Knust, 2019; Aguilar-Aragon et al., 2020). Crb is correctly localized by Rab11-containing endosomes using motor-driven transport along polarized microtubules and F-actin filaments. Interestingly, upon loss of microtubule minus-end director protein dynein, Rab11 endosomes containing Crb are transported basally rather than apically (Aguilar-Aragon et al., 2020). Once Crb is successfully addressed to the apical membrane, it is delivered at the correct localization on the plasma membrane using exocyst-mediated delivery. Mutant clones for sec15 or sec5, subunits of the exocyst, strongly disrupts the apical localization of Crb, confirming the essential requirement of the exocyst in delivery of Crb to the apical membrane (Aguilar-Aragon et al., 2020). In cultured mammalian cells, the exocyst associates with adherens junctions and PAR3 (Ahmed and Macara, 2017; Polgar and Fogelgren, 2018). Because of the known interaction between CRB, PAR6, and PAR3 in mammals, mentioned below, this trafficking model might be conserved among species. Further investigations are required to test whether the mechanisms of epithelial polarization are conserved in humans.

## CRB Protein Function in Mammalian Tissues

Various research studies have shown that CRB1 and CRB2 are apical polarity factors, and apical-basal cell polarity is essential for the formation and function of epithelial tissues (Bazellières et al., 2018). More research is required to define the function of the recently described CRB1-B isoform because of its distinct 5′ and 3′ domain. Below, we will describe the canonical function of CRB in maintaining cell adhesion and morphogenesis, and its role in cell division and development.

### Maintaining Cell Adhesion and Morphogenesis

**FIGURE 2:**
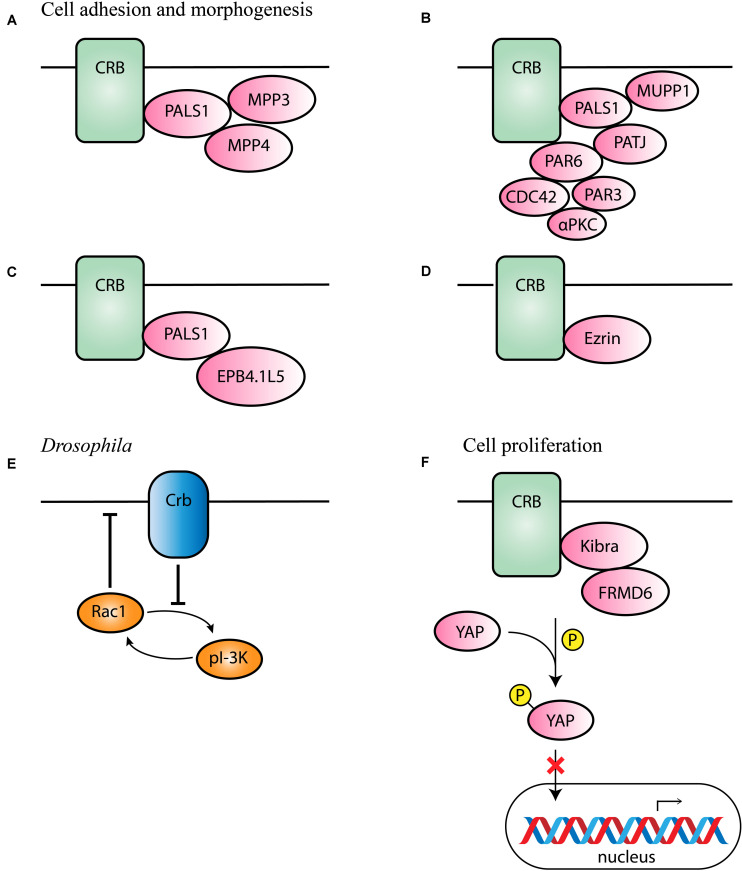
Schematic representation of CRB interaction partners. **(A–D)** Proposed interaction partners and formed CRB complexes in mammals involved in cell adhesion and morphogenesis. **(E)** Proposed interaction partner in *Drosophila* suggested to be conserved among species. **(F)** Proposed interaction partners involved in cell proliferation.

The prototypic ERLI sequence of CRB proteins is important for interaction with key adaptor proteins. The core CRB complex is formed by interaction of CRB and protein associated with Lin Seven 1 (PALS1), also known as membrane-associated guanylate kinase p55 subfamily member 5 (MPP5), where PALS1 binds to the conserved C-terminal PDZ domain of CRB (Roh et al., 2002; van de Pavert et al., 2004; Margolis, 2018). Ablation of *Mpp5* in mouse RPE causes early onset retinal degeneration, whereas ablation of *Mpp5* in the neural retina does not, suggesting an essential role of PALS1 at the tight junctions of RPE but not in the neural retina (Park et al., 2011). The core CRB complex is evolutionary conserved and regulates apical-basal polarity and maintains cell adhesion (Bulgakova and Knust, 2009). Pals1 can interact with Mpp3 and Mpp4 at the SAR in the mouse retina (Figure 2A; Kantardzhieva et al., 2005, 2006; Dudok et al., 2013). *Mpp3* conditional knockout (cKO) mice with Mpp3 specifically ablated in the retina showed disrupted localization and reduced levels of Pals1, indicating that Mpp3 is essential to maintain levels of Pals1 at the SAR near the outer limiting membrane (Dudok et al., 2013).

Additionally, binding of PALS1 and CRB can lead to the recruitment of PATJ or multiple PDZ domain protein 1 (MUPP1) to the apical membrane (Figure 2B; Roh et al., 2002). PATJ connects and stabilizes apical and lateral components of tight junctions in human intestinal cells (Michel et al., 2005). In some cells, both MUPP1 and PATJ complexes co-exist, and MUPP1 regulates the cellular levels of the PALS1/PATJ polarity complex (Assémat et al., 2013). Co-immunoprecipitation studies showed that Mupp1 interacts in the mouse retina with Crb1 and Pals1, but not or less with Patj (van de Pavert et al., 2004). PATJ preferentially binds with PALS1 and partitioning defective-6 homolog (PAR6; Figure 2B; Adachi et al., 2009; Assémat et al., 2013). PAR6 is a key adaptor protein that interact with the ERLI PDZ domain of CRB in mammalian cells (Hurd et al., 2003; Lemmers et al., 2004). PAR6 leads to recruitment of PAR3, atypical protein kinase C (αPKC) and cell division control 42 (CDC42), known as the PAR complex (Joberty et al., 2000; Lin et al., 2000; Suzuki et al., 2001; Yamanaka et al., 2001; Hurd et al., 2003; Whitney et al., 2016; Pichaud, 2018). This PAR6-CDC42 complex is required for the apical-basal polarity and cell adhesion (Yamanaka et al., 2001).

Alternatively, the importance of a CRB-PALS1-EPB4.1L5 complex in mammals has been described (Laprise et al., 2006, 2009; Gosens et al., 2007). Co-expression and co-localization studies suggested that in several epithelial derived tissues Epb4.1l5 interact with at least one Crumbs homolog and with PALS1 (Figure 2C). In addition, in the adult retina, Epb4.1l5 showed substantial overlap at the outer limiting membrane (OLM) with CRB1 and PALS1 (Gosens et al., 2007). Overexpression of Epb4.1l5 in polarized MDCK cells affects tightness of cell junctions and results in disorganization of the tight junction markers ZO-1 and PATJ (Gosens et al., 2007). However, another group discovered, unlike in the zebrafish and *Drosophila* orthologs, that mouse Crumbs proteins are localized normally in absence of Epb4.1l5 (Lee et al., 2007). Additional research should be performed to define its precise molecular mechanism in mammalian cells.

The alternative isoform CRB3B contains a distinct carboxy terminal motif namely the CLPI motif, suggesting different binding partners in epithelial cells. CRB3 is widely expressed in epithelial cells. A *Crb3* KO mouse demonstrates extensive defects in epithelial morphogenesis, the mice die shortly after birth with cystic kidneys and lung proteinaceous debris throughout the lungs (Whiteman et al., 2014). Interestingly, these defects are also seen in Ezrin knockout mice, which is in line with the detected interaction between CRB3B and Ezrin in mice and mammalian cells (Figure 2D; Whiteman et al., 2014; Tilston-Lünel et al., 2016). This indicates that CRB3B is also crucial for epithelial morphogenesis and plays a role in linking the apical membrane to the underlying cytoskeleton (Whiteman et al., 2014; Gao et al., 2016; Tilston-Lünel et al., 2016). Therefore, the roles of the two CRB3 variants in the mouse lungs remain to be determined.

D*rosophila* Crb has also been found to inhibit the positive-feedback loop of phosphoinositide 3-kinase (PI-3K) and Rac1, thereby repressing the activation of Rac1 as well as PI-3K and maintaining proper apical domain and epithelial tissue integrity (Figure 2E; Chartier et al., 2011). This process could potentially be conserved in different species, however, more research in mammalian cells is required to support this hypothesis.

### CRB Function in Cell Proliferation

In MGCs of *Drosophila* and *Xenopus*, yes-associated protein 1 (YAP) an important role in damaged retina (Hamon et al., 2017, 2019; Rueda et al., 2019). YAP is a core member of the Hippo pathway, which regulates several biological processes including cell proliferation, and survival (Yu et al., 2015). The intracellular domain of *Drosophila* Crb interacts with Expanded and thereby regulates the activity of Hippo pathway kinases (Yu and Guan, 2013; Yu et al., 2015). In mammalian lung epithelial and breast cancer cells, CRB3 expression also correlates with the Hippo pathway (Szymaniak et al., 2015; Mao et al., 2017). More specifically, CRB3 affects the Hippo pathway by interacting with Kibra and/or FRMD6 (FRMD6 is the homolog of *Drosophila* Expanded; Figure 2F). With low CRB3 expression levels, the Hippo-pathway is inactivated, YAP is not phosphorylated and can move to the nucleus where YAP target genes are expressed leading to increased cell proliferation and decreased apoptosis (Mao et al., 2017). Also in *Crb1*^rd8^ versus wild-type mouse retina several Hippo signaling related genes were differentially expressed (Hamon et al., 2017). In addition, CRB1 and CRB2 deletion also lead to YAP signaling dysregulation in developing murine retinas (Pellissier et al., 2013). These data suggest an essential role of the Hippo pathway in the control of cell proliferation.

## CRB1 and CRB2 in Retinal Diseases

Mutations in the *CRB1* gene are associated with a wide spectrum of retinal dystrophies, such as retinitis pigmentosa (RP) and Leber congenital amaurosis (LCA). RP is a clinically and genetically heterogeneous disease affecting more than 1.5 million people worldwide, where patients typically experience night blindness followed by progressive visual field loss ultimately leading to complete loss of vision in early or middle-life (Talib et al., 2017; Verbakel et al., 2018). The age at symptom onset for RP patients ranged from 0 to 47 years, with a median onset of 4 years (Talib et al., 2017). Approximately 3–9% of non-syndromic cases of autosomal recessive RP are caused by a mutation in the *CRB1* gene (Vallespin et al., 2007; Bujakowska et al., 2012; Corton et al., 2013). LCA is a more severe retinal dystrophy, causing serious visual impairment or blindness in newborns (Den Hollander et al., 2004). Mutations in the *CRB1* gene account for approximately 7–17% of all LCA cases (Den Hollander et al., 2004; Vallespin et al., 2007; Bujakowska et al., 2012; Corton et al., 2013). There are more than 200 different mutations described along the *CRB1* gene resulting in retinal dystrophies without a clear genotype-phenotype correlation with RP or LCA, *CRB1* patients may display unique clinical features such as pigmented paravenous chorioretinal atrophy, macular atrophy alone, retinal degeneration associated with Coats-like exudative vasculopathy, para-arteriolar preservation of the RPE, or nanophthalmia (Henderson et al., 2011; Bujakowska et al., 2012; Corton et al., 2013). The clinical variability of disease onset and severity, even within a patient cohort with the same homozygous mutations, supports the hypothesis that the phenotype of patients with *CRB1* mutations is modulated by other factors (Mathijssen et al., 2017). So far, no treatment options exist for patients with mutations in the *CRB1* gene. To study *CRB1*-related retinal dystrophies for treatment options, several models have been used. Below, *CRB1-*related human- and animal-derived retinal models are described.

Until recently, no RP patients were described with mutations in the *CRB2* gene. However, Chen et al. (2019) discovered, using whole exome sequencing (WES), a homozygous *CRB2* p.R1249G mutation in a consanguineous Chinese family presenting RP. This mutation disturbs the stability of CRB2 protein and thereby induces RPE degeneration, impairs RPE phagocytosis, and accelerates RPE apoptosis. However, only a limited number of patients with this mutation are described, identification of *CRB2* mutations in more RP patients is warranted to better support its pathogenicity.

## Human-Derived Retinal Models

The use of human-induced pluripotent stem cell (hiPSC) models for research is an emerging strategy to explore patient phenotypes *in vitro.* These techniques allow access to previously limited or inaccessible material and have been explored in many ophthalmic laboratories worldwide. A commonly used method is the differentiation of hiPSC into retinal organoids. Since the first one, numerous groups have adapted or created their own method to more efficiently generate well laminated retinal organoids (Meyer et al., 2011; Nakano et al., 2012; Zhong et al., 2014; Luo et al., 2018; Ovando-Roche et al., 2018). Nevertheless, a wide variability in differentiation efficiency across hiPSC lines is often reported (Capowski et al., 2019; Cora et al., 2019; Mellough et al., 2019; Chichagova et al., 2020). Chichagova et al. (2020) have shown that the ability of three different hiPSC lines to differentiate into retinal organoids in response to IGF1 or BMP4 activation was line- and method-dependent. Hallam et al. (2018) differentiated five hiPSC lines with a variability in efficiency, but by 5 months of differentiation all the retinal organoids were able to generate light responses and contained a well-formed ONL with PRCs containing inner segments, cilia, and outer-like segments. Attempts have been made to decrease this wide variability, Luo et al. (2018) described that the use of a Wnt signaling pathway antagonist, Dickkopf-related protein 1 (DKK-1), efficiently generated retinal organoids in all six hiPSC lines. Bulk RNA-sequencing profiling of retinal organoids demonstrated that the retinal differentiation *in vitro* recapitulated the *in vivo* retinogenesis in temporal expression of cell differentiation markers, retinal disease genes, and mRNA alternative splicing (Kim et al., 2019). These results make the retinal organoids, despite their high variability, of great interest in a wide range of applications including drug discovery, investigating the mechanism of retinal degeneration, developing cell-based therapeutic strategies and many more.

Defining the localization and onset of expression of the CRB complex members has been achieved in healthy hiPSC-derived retinal organoids. Several members of the CRB complex, CRB2, PALS1, PATJ, and MUPP1, were detected at the outer limiting membrane as early as differentiation day 28 (DD28), typical and clear puncta-like staining patterns for CRB1 were found only after DD120. All CRB complex members together with adherence junction markers, p120-catenin and N-cadherin, were still detectable in DD180 retinal organoids (Quinn et al., 2019a). The onset of CRB1 and CRB2 protein expression recapitulates those observed in the human fetal retina, with a clear onset of CRB2 expression before CRB1 expression (Quinn et al., 2019a).

Three *CRB1* patient hiPSC lines containing a homozygous missense mutation (c.3122T > C), or heterozygous missense mutations (c.2983G > T and c.1892A > G, or c.2843G > A and c.3122T > C) were successfully differentiated into retinal organoids and analyzed at DD180. Here, all three retinal layers were developed: retinal ganglion cell layer marked by Tuj1 positive dendrites, neuroblast layer marked by SOX9 positive retinal progenitor cells, and an outer nuclear layer marked by recoverin positive PRCs. However, frequently, there were ectopic recoverin positive cells found above the outer limiting membrane and all missense *CRB1* organoid lines developed small but frequent disruptions of localization of CRB complex members at the OLM that were not found in control lines (Quinn et al., 2019a). Data from these *CRB1* patient hiPSC retinal organoids suggest a retinal degeneration phenotype similar to that previously found in mice lacking CRB1, mice expressing the C249W CRB1 variant, or mouse retina lacking CRB2 (van de Pavert et al., 2004, 2007a; Alves et al., 2013). Another study shortly describes the successful differentiation of hiPSC carrying a compound heterozygous mutation in the *CRB1* gene (c.1892A > G and c.2548G > A) to retinal organoids. All three germ layers and expressed markers of retinal progenitor cells, including N-cadherin, rhodopsin, and PAX6, after 35 days of differentiation were present, but no phenotype was described in this paper (Zhang et al., 2018). To our knowledge, only these two papers have reported the generation of *CRB1* patient hiPSC-derived retinal organoids. The reproducible phenotype observed in these three *CRB1* patient lines (Quinn et al., 2019a) provides a good model for assessing potential gene therapy approaches.

Another frequently used method in the ophthalmic field is the differentiation of hiPSC into RPE monolayers. Efficient protocols for differentiating hiPSC into RPE monolayers using a mixture of growth factors have been established (Zahabi et al., 2012; Buchholz et al., 2013; Shutova et al., 2016). However, attempts are currently made to use non-biological products, such as small molecules, that would limit the risks of infection or immune rejection when transplanted. Maruotti et al. (2015) developed a protocol which uses chemotin (CTM) in combination with a previously known neural inducer nicotinamide (NIC) to efficiently differentiate hiPSC into RPE monolayers. In three independent hiPSC lines, RPE differentiation was efficient, and key RPE markers such as microphthalmia-associated transcription factor (MITF), PMEL17, and tight junction protein zonula occludens 1 (ZO-1) were strongly expressed. When left longer in culture to mature, bestrophin 1 (BEST1) and RPE-specific protein of 65 kDa (RPE65) were also strongly expressed (Maruotti et al., 2015). Using a slightly adapted protocol, Smith et al. (2019) differentiated six more hiPSC lines into hiPSC-RPE monolayers, all six also expressed the key RPE markers ZO-1, BEST-1, and MITF. Another study revealed by RNA sequencing data that hiPSC-RPE grouped with fetal RPE samples, indicating that their gene expression was highly correlated and similar (Smith et al., 2019). In addition, Zahabi et al. (2012) provided proof-of-concept that multiple retinal-disease specific hiPSC lines, including two RP lines, can be differentiated into RPE monolayers. Altogether, this data illustrates the potential of hiPSC-RPE as a model system for retinal diseases with mutations in the RPE. Related to CRB, recent studies have shown that CRB2 but not CRB1 is expressed in the human RPE during the differentiation into retinal organoids (Quinn et al., 2019a). In addition, there are RP patients described with specific *CRB2* variations expressed in RPE cells (Chen et al., 2019). Therefore, the method of generating hiPSC-RPE could be used to explore treatment possibilities for patients with specific variations in *CRB2*.

## *CRB1*-Related Animal Retinal Degeneration Models

Numerous research groups focus on animal models to gain, understand, and develop gene therapy strategies that potentially can be used to treat retinal degeneration of RP and LCA patients. Over the years there are multiple animal models developed mimicking the CRB1-related phenotype in patients. These models vary from mild to more severe, early- to late-onset, and MGC or photoreceptor specific phenotypes. Double retinal knock-outs of *CRB1* and *CRB2*, have helped to understand the contribution of the two CRB proteins to the retinal disease etiology, and explain the relatively mild phenotype observed in *Crb1* variant mouse models (Mehalow et al., 2003; van de Pavert et al., 2004, 2007a,b).

### LCA-Like Mouse Models

Four mouse models showing a *CRB1* LCA-like phenotype have been reported: *Crb1*^KO^*Crb2*^ΔRPC^ where both Crb1 and Crb2 are ablated in retinal progenitor cells (Pellissier et al., 2013), secondly the *Crb1*^KO^*Crb2*^ΔimPRC^ where Crb1 is ablated in MGC and Crb2 is ablated in immature PRCs with remaining Crb2 levels in MGC and progenitor cells (Quinn et al., 2018), thirdly the *Crb1*^KO/WT^*Crb2*^ΔRPC^ mouse model with reduced levels of Crb1 in MGC and ablation of Crb2 in retinal progenitor cells (Pellissier et al., 2013), and finally *Crb1*^KO^*Crb2*^ΔMGC^ in which both Crb1 and Crb2 are ablated in MGC (Quinn et al., 2019b). All four models exhibit vision loss indicated by a reduced electroretinography (ERG) response. In addition, retinal degeneration was observed by outer limiting membrane disruptions, abnormal retinal lamination, intermingling of nuclei of the ONL and INL, and ectopic localization of retinal cells. These mouse models show an early onset phenotype with distinct severity indicated by the order mentioned above. In short, in the most severe mouse model, *Crb1*^KO^*Crb2*^ΔRPC^, the phenotype onset was found as early as embryonal day 13 (E13) which was observed throughout the retina (Pellissier et al., 2013). Retinal degeneration in *Crb1*^KO^*Crb2*^ΔimPRC^ was detected at E15 in the whole retina, but in adult mice the superior retina was more affected than the inferior retina (Quinn et al., 2018). Also in *Crb1*^KO/WT^*Crb2*^ΔRPC^ mice retinal degeneration was detected at E15, but was mostly affecting the peripheral retina (Pellissier et al., 2013). Finally, the *Crb1*^KO^*Crb2*^ΔMGC^ mice showed the first signs of degeneration at E17, where mostly the peripheral retina was affected. More subtle differences between these models are described and summarized before (Quinn et al., 2019b). These data show that all four *Crb1* mouse models mimic the LCA phenotype in patients and could therefore be used for future therapy development.

### RP-Like Mouse Models

Twelve *CRB1* RP-like mouse models have been described so far, including (1) *Crb1*^KO/C249W^ with a missense variation in the *Crb1* gene (van de Pavert et al., 2007a), (2) *Crb2*^ΔMGC^ where only Crb2 is ablated in MGC (Alves et al., 2014), (3) the *Crb1*^rd8^ mice with a naturally occurring nonsense mutation in the *Crb1* gene (Mehalow et al., 2003), (4) the *Crb1*^KO^ where the full length Crb1 protein is ablated from retinal radial glial progenitor cells, MGCs, and the rest of the body (van de Pavert et al., 2004), (5) the *Crb1*^del–B^ were *Crb1-B* is abolished from photoreceptor and MGCs (Ray et al., 2020), (6) the *Crb1*^null^ where a deletion of alternate exon 5a up to intron 7 disrupts all *Crb1* isoforms (Ray et al., 2020), (7) *Crb2*^Δrods^ where Crb2 is ablated only in developed rod PRCs (Alves et al., 2019), (8) *Crb1*^KO^*Crb2*^Δrods^ where Crb1 is ablated in MGC and Crb2 in rod PRCs (Alves et al., 2019), (9) *Crb1*^KO^*Crb2*^low–imPRC^ with absence of Crb1 and reduced levels of Crb2 in immature photoreceptors (Quinn et al., 2018), (10) *Crb1*^KO^*Crb2*^Δlow–RPC^ with absence of Crb1 and reduced levels of Crb2 in retinal progenitor cells (Pellissier et al., 2014b), (11) *Crb2*^ΔRPC^ where Crb2 is ablated in retinal progenitor cells (Alves et al., 2013), and finally, (12) *Crb2*^ΔimPRC^ with Crb2 ablation in immature PRCs (Alves et al., 2014). The different Cre mouse models show variations in mosaicism (mutant adjacent to wildtype cells), but eleven out of twelve *Crb* models show disruptions at the outer limiting membrane with rows or single photoreceptor nuclei protruding into the subretinal space or ingressing into the outer plexiform layer leading to a degenerative retinal phenotype (Table 1).

**TABLE 1:** Schematic overview retinitis pigmentosa (RP) mouse models.

	*Crb1*^KO/C249W^	*Crb2*^Δ^^MGC^	*Crb1*^del–B^	*Crb1*^rd8^	*Crb1*^KO^	*Crb1*^null^	*Crb2*^ΔRods^	*Crb1*^KO^ *Crb2*^ΔRods^	*Crb1*^KO^ *Crb2*^low–imPRC^	*Crb1*^KO^ *Crb2*^low–RPC^	*Crb2*^ΔRPC^	*Crb2*^ΔimPRC^
Severity	+	+	−	++	++	++	++	+++	+++	+++	++++	++++
CRB1-A ablation	MGC	−	−	Natural occurring mutation	MGC	MGC	−	MGC	MGC	MGC	−	−
CRB1-B ablation	−	−	PRC and MGC	Natural occurring mutation	−	PRC and MGC	−	−	−	−	−	−
CRB2 ablation	−	MGC	−	−	−	−	Rod PRC	Rod PRC	50% in immature PRC	50% in RPC	RPC	Immature PRC
Morphologic phenotype onset^∗^	8M	1M	NA	1M	P14	3M	3M	1M	P10	P10	E18.5	E15.5
ERG differences^∗^	No	No	NA	No	No	NA	Yes (9 M)	Yes (3 M)	Yes (3 M)	Yes (3 M)	Yes (1 M)	Yes (1 M)
Affected areas	NA	Mainly periphery	NA	Inferior nasal quadrant	Inferior temporal quadrant	Inferior retina	Superior retina	Superior retina	Inferior retina	Throughout retina	Throughout retina	Throughout retina
OLM disruptions	Yes, sporadic	Yes	No	Yes	Yes	Yes	Yes	Yes	Yes	Yes	Yes	Yes
PRC nuclei protrusions	Yes, sporadic	Yes	No	Yes	Yes	Yes	Yes	Yes	Yes	Yes	Yes	Yes

*MGC, Müller glial cells; OLM, outer limiting membrane; PRC, photoreceptor cell; RPC, retinal progenitor cell. ^∗^ The phenotype onset and ERG differences can be marginal on the indicated ages, the severity of the phenotype increases over time.*

The *Crb1*^rd8^ mice have a naturally occurring single base deletion in exon 9 of the *Crb1* gene causing a frame shift and premature stop codon, thereby truncating the transmembrane and cytoplasmic domain of Crb1. This results in a photoreceptor degeneration mainly observed in the inferior nasal quadrant of the eye, caused by retinal folds and pseudorosettes (Mehalow et al., 2003). In the *Crb1*^KO^ mouse model the retinal lamination is predominantly maintained, and degeneration is found in the inferior temporal quadrant of the retina. Degeneration is indicated by single or groups of PRCs protruding into the subretinal space, rosette formation, and neovascularization. In 18M-old mice there was no loss of overall retinal function measured by electroretinography, suggesting that a major part of the retina was not affected by loss of Crb1 (van de Pavert et al., 2004, 2007b). Light exposure experiments reveal that light exposure doesn’t initiate but rather enhances the retinal degeneration (van de Pavert et al., 2007b). The mild phenotype observed in these *Crb1* mouse models suggests that Crb2 protein may compensate the effect of CRB1 deletion.

In the *Crb1*^del–B^ the alternative *Crb1-B* isoform is abolished from PRCs, while the *Crb1*^null^ disrupts all potential *Crb1* isoforms (Ray et al., 2020). The *Crb1*^del–B^ mouse do not show significant disruptions in the OLM, while the disruptions in the *Crb1*^null^ mice were comparable with *Crb1*^rd8^ (Ray et al., 2020). Although not mentioned by Ray et al. (2020), also the *Crb1*^KO^ mice shows significant OLM disruptions strongly depending on genetic background as well as exposure to light (van de Pavert et al., 2004, 2007b). As the IrCaptureSeq suggested that *Crb1-B* is the most abundant transcript in mouse and human retina, a cross-breeding of *Crb1*^null^ with *Crb1*^del–B^ mice was performed to define the relevance of Crb1-B in the retina. This heterozygous *Crb1*^null/del–B^ showed similar OLM disruptions with the homozygous *Crb1*^null^ mouse (Ray et al., 2020). Similar OLM disruptions were thus found in *Crb1*^rd8^, *Crb1*^null^, *Crb1*^null/del–B^, and *Crb1*^KO^ mice. It is essential to perform retinal function measurements on the mouse models affecting *Crb1-B* to understand its function and to compare it with previously described *Crb1* mouse models. In addition, the most severe retinal phenotype so far derives from *Crb1* mice with concomitant loss of Crb2. Both *Crb2*^ΔRPC^ and *Crb2*^ΔimPRC^ show an early onset retinal degeneration at embryonic day 18.5 (E18.5) and E15.5, respectively, the difference is caused by the distinct expression pattern and timing of the Cre recombinase and morphological phenotypes result in differences in the scotopic and photopic ERG conditions already at 1 M of age (Alves et al., 2013, 2014). Interestingly, in contrast to *Crb2*^ΔRPC^ mice, the lamination of Müller glial, ganglion and amacrine cells were also misplaced in *Crb2*^ΔimPRC^ mice. These lamination defects were also observed in *Crb1*^KO/WT^*Crb2*^ΔRPC^ mice (Pellissier et al., 2013).

Another mouse model, *Crb2*^Δrods^, show a mild and late onset phenotype limited to the superior retina (Alves et al., 2019). In some 3 M and all 6 M old *Crb2*^Δrods^ mice, disruptions of the outer limiting membrane, a thinned photoreceptor layer at the peripheral superior retina, and PRCs protruding into the subretinal space were observed. A reduction in ERG scotopic a-wave was observed in 9 M old mice, while no difference in optokinetic head tracking response (OKT) spatial frequency or contrast sensitivity was observed. Interestingly, while most PRCs were affected, all the remaining PRCs showed mature inner- and outer-segments. Remaining rods were functional and the cones showed normal morphology. This phenotype is enhanced by a concomitant loss of Crb1 in MGCs (Alves et al., 2019). These mice, *Crb1*^KO^*Crb2*^Δrods^, show a similar but enhanced phenotype in comparison to *Crb2*^Δrods^ mice. Here, a reduction in ERG scotopic a-wave was observed at 3 M and became more apparent at 6 M onward. In addition, a significant decrease in OKT contrast sensitivity was found from 3 M of age onward (Alves et al., 2019). The difference in phenotype onset between all these mouse models might be explained by the onset of Cre expression during retinogenesis and by cell-type specific ablation; ablation of Crb2 from a later time point resulted in a milder phenotype. Interestingly, when Crb2 is ablated in MGCs, *Crb2*^ΔMGC^, a very mild morphological phenotype with no functional consequences measured using ERG was observed (Alves et al., 2014), but ablation of both Crb1 and Crb2 from MGCs caused a severe LCA phenotype (Quinn et al., 2019b). This data suggests that Crb2 has a redundant function in MGCs, while in PRCs it is essential for proper retinal lamination and function.

Interestingly, the *Crb1*^KO^ phenotype is located at the inferior temporal quadrant whereas the *Crb2*^Δrods^ phenotype is mainly observed at the peripheral and central superior retina. These differences might be related to higher levels of Crb2 in the inferior retina while Crb1 is expressed at higher levels in the superior retina (Pellissier et al., 2014b). In addition, there might be modifying factors present which are enriched in either the superior or inferior retina causing the different phenotypes.

In addition to these mouse models, a rat with a spontaneous mutation in *Crb1* exon 6 was discovered mimicking human macular telangiectasia type 2 (Zhao et al., 2015). The autosomal recessive indel mutation causes an early onset phenotype with a strongly reduced ERG response in 3-week old rats. These rats showed a focal loss of retinal lamination, OLM disruptions, and PRC, MGC, and RPE alterations (Zhao et al., 2015). Differences between this and the *Crb1* mouse models could result from different types of mutations or from different genetic setups displayed by these different animal species.

## Gene Augmentation Strategies for *CRB1* Retinal Dystrophies

There is an emerging interest in gene augmentation strategies for retinal dystrophies. Recently, gene therapy became available for young RP and LCA patients with biallelic mutations in the *RPE65* gene. Voretigene neparvovec-rzyl, or its commercial name: LUXTURNA^TM^, uses the adeno-associated viral vector serotype 2 (AAV2) to deliver by subretinal injection a functional copy of the *RPE65* gene into the RPE cells. *RPE65* transgene expression results in the production and correct localization of RPE65 protein in RPE cells, thereby compensating for the loss of the protein and restoring the visual cycle in these patients (Maguire et al., 2019). Nowadays, there are numerous clinical studies ongoing which explore the use of AAV as a therapeutic vector for retinal diseases, such as RP, wet age-related macular dystrophy (AMD), LCA, and many more (Wang et al., 2019). However, so far, no treatment options are available for RP and LCA patients with mutations in the *CRB1* gene. Below, we will describe novel therapeutic tools which could be promising for *CRB1* retinal gene augmentation therapy in RP patients.

Currently, AAVs are the leading platform for gene delivery in the treatment of retinal dystrophies. AAVs are mainly investigated because of their low toxicity, their capability to transduce both dividing and non-dividing cells, they do not integrate into the host genome, and AAV capsid variants display distinct cell tropisms. A complete overview of basic AAV biology, AAV vectorology, and current therapeutic strategies and clinical progress was recently reviewed by Wang et al. (2019) and Buck and Wijnholds (2020). The major disadvantage of AAVs is their limited package capacity, bigger gene expression cassettes than 4.5 kb are not able to fit within a single AAV. Because of this, the development of AAV-mediated *CRB1* gene therapy is also challenging. Full-length cytomegalovirus (CMV) ubiquitous promoter and *CRB1* cDNA exceeds the AAV package limitation. However, using an engineered minimal CMV promoter and codon optimized *CRB1* cDNA allowed sufficient expression levels of full-length CRB1 protein in MGCs in mice (Pellissier et al., 2014a). Pre-clinical studies in mouse using this AAV-CMVmin-*hCRB1* have shown that expression of *CRB1* was deleterious in *CRB1* mouse models but not in wild-type mice (Pellissier et al., 2015). In addition, there are more potential *CRB1* transcript variants which could be targeted (Quinn et al., 2017). Interestingly, alternative strategies using the codon-optimized structural and functional family member *CRB2* to rescue *CRB1*-related retinopathies restored retinal function and structure in *Crb1* mouse models (Pellissier et al., 2015). In that study, improved photoreceptor layer morphology and ERG response was detected after *CRB2* delivery. The combination of AAV9 with a full-length CMV promoter was used to target both photoreceptor and MGCs, whereas no rescue was observed when either photoreceptor or MGCs were targeted with *CRB2* (Pellissier et al., 2015). In addition, several groups explored the possibility to overcome the limiting AAV package capacity by dual or triple-AAV approaches (Trapani et al., 2014; Carvalho et al., 2017; Maddalena et al., 2018) or by increasing the package capacity (Ding and Gradinaru, 2020). Yet, the studies performed so far have shown that the dual AAV approaches have lower expression levels compared to a single AAV vector (Trapani et al., 2014; Carvalho et al., 2017).

Several research groups focus on AAV-mediated gene delivery to define the tropism in hiPSC-derived retinal organoids and/or RPE. Out of four different AAV capsids packaged with the CAG promoter and GFP (AAV2, AAV2-7m8, AAV8, AAV9), Garita-Hernandez et al. (2020) showed with AAV2-7m8 infection at day 44 the most efficient transduction of the hiPSC-derived retinal and RPE organoids. Limited transduction of AAV9 was found (Garita-Hernandez et al., 2020). Another study showed, using AAV-CMV-*GFP* constructs, a more efficient retinal organoid transduction at DD220 especially in MGC with AAV5 or ShH10Y445F capsids in comparison with AAV9 (Quinn et al., 2019a). Recently, Lane et al. (2020) demonstrate efficient PRCs transduction in DD140 retinal organoids using an AAV5 packaged with the CAG promoter. Moreover, AAV5-mediated gene augmentation with human *RP2* was able to rescue the degeneration found in *RP2* KO retinal organoids by preventing ONL thinning and restoring rhodopsin expression (Lane et al., 2020). Therefore, testing CRB1- or CRB2-expressing vectors in retinal organoids will aid in the discovery of *CRB* gene therapy treatment for *CRB1-*related retinal dystrophies in patients.

In addition, there are different non-viral mediated approaches for gene therapy. An example is the use of nanoparticles. Three representative nanoparticles, namely metal-based, polymer-based, and lipid-based nanoparticles, were recently reviewed describing their characteristics and recent application in ocular therapy (Wang et al., 2018). Shortly, the most extensive characterized nanoparticle is the polymer-based CK30-PEG nanoparticle, which contains plasmid DNA compacted with polyethylene glycol-substituted lysine 30-mers (Fink et al., 2006; Han et al., 2012). Theoretically, these approaches have an unlimited gene packaging capacity, which display a big advantage for large genes such as *CRB1.* These nanoparticles are also able to infect RPE and PRC, and can drive gene expression on a comparable scale and longevity than AAVs in mice (Cai et al., 2009; Han et al., 2012). CK30-PEG nanoparticles have a tolerable safety profile and is non-toxic in mouse and non-human primate eyes (Ding et al., 2009; Akelley et al., 2018). To our knowledge, there is no successful clinical study described using these nanoparticles so far. More research is required to define the clinical relevance of these nanoparticles in retinal gene therapy. Further pre-clinical research and clinical trials will aid in the discovery of new gene therapy approaches for *CRB1-*related retinal dystrophies in patients. A retrospective *CRB1* natural history study (NHS) has been published (Talib et al., 2017), and their prospective NHS with strategies for evaluation of the efficacy of novel clinical therapeutic interventions in the treatment of *CRB1*-retinal dystrophies will be reported in the end of 2020. Recently, a French biotech company called HORAMA signed an exclusive license agreement with Leiden University Medical Center targeting *CRB1* gene mutations to treat inherited retinal dystrophies. Based on current timelines, HORAMA expects initiating a Phase I/II clinical study with the drug candidate in 2023 (HORAMA, 2020).

## Conclusion

In this review we have discussed (1) CRB protein function in mammalian cells, (2) recent advances and potential tools for *CRB1* human and animal retinal degeneration models, and (3) described therapeutic tools which potentially could be used for retinal gene augmentation therapy for RP and LCA patients with mutations in the *CRB1* gene.

## Author Contributions

NB, JW, and LP contributed to writing, reviewing, and editing the manuscript. NB wrote the original draft. All authors contributed to the article and approved the submitted version.

## Conflict of Interest

The LUMC is the holder of patent number PCT/NL2014/050549, which describes the potential clinical use of *CRB2*; JW and LP are listed as co-inventors of this patent, and JW is an employee of the LUMC. The remaining author declares that the research was conducted without any commercial or financial relationship that could be construed as a potential conflict of interest.

## Footnotes

1 http://grch37.ensembl.org/Homo_sapiens/Gene/Summary?db=core;g=ENSG00000134376;r=1:197170592-197447585
